# Integrated gene and miRNA expression analysis of prostate cancer associated fibroblasts supports a prominent role for interleukin-6 in fibroblast activation

**DOI:** 10.18632/oncotarget.5056

**Published:** 2015-09-08

**Authors:** Valentina Doldi, Maurizio Callari, Elisa Giannoni, Francesca D'Aiuto, Massimo Maffezzini, Riccardo Valdagni, Paola Chiarugi, Paolo Gandellini, Nadia Zaffaroni

**Affiliations:** ^1^ Department of Experimental Oncology, Fondazione IRCCS Istituto Nazionale dei Tumori, 20133, Milan, Italy; ^2^ Department of Experimental and Clinical Biomedical Sciences, University of Florence, 50134, Florence, Italy; ^3^ Department of Urology, Fondazione IRCCS Istituto Nazionale dei Tumori, 20133, Milan, Italy; ^4^ Department of Radiation Oncology 1, Fondazione IRCCS Istituto Nazionale dei Tumori, 20133, Milan, Italy; ^5^ Prostate Program, Fondazione IRCCS Istituto Nazionale dei Tumori, 20133, Milan, Italy

**Keywords:** prostate cancer, cancer associated fibroblasts, interleukin-6, microRNA, gene expression

## Abstract

Tumor microenvironment coevolves with and simultaneously sustains cancer progression. In prostate carcinoma (PCa), cancer associated fibroblasts (CAF) have been shown to fuel tumor development and metastasis by mutually interacting with tumor cells. Molecular mechanisms leading to activation of CAFs from tissue-resident fibroblasts, circulating bone marrow-derived fibroblast progenitors or mesenchymal stem cells are largely unknown. Through integrated gene and microRNA expression profiling, we showed that PCa-derived CAF transcriptome strictly resembles that of normal fibroblasts stimulated *in vitro* with interleukin-6 (IL6), thus proving evidence, for the first time, that the cytokine is able *per se* to induce most of the transcriptional changes characteristic of patient-derived CAFs. Comparison with publicly available datasets, however, suggested that prostate CAFs may be alternatively characterized by IL6 and TGFβ-related signatures, indicating that either signal, depending on the context, may concur to fibroblast activation. Our analyses also highlighted novel pathways potentially relevant for induction of a reactive stroma. In addition, we revealed a role for muscle-specific miR-133b as a soluble factor secreted by activated fibroblasts to support paracrine activation of non-activated fibroblasts or promote tumor progression.

Overall, we provided insights into the molecular mechanisms driving fibroblast activation in PCa, thus contributing to identify novel hits for the development of therapeutic strategies targeting the crucial interplay between tumor cells and their microenvironment.

## INTRODUCTION

Tumor microenvironment is a dynamic and heterogeneous system of untransformed cells, growth factors, cytokines and pro-angiogenic molecules that coevolves with cancer progression [1]. The most representative nonmalignant stromal cells in the cancer microenvironment are fibroblasts, mesenchymal cells of the connective tissue that are embedded within the extra-cellular matrix (ECM). In normal epithelia, fibroblasts are responsible for tissue architecture, function and homeostasis by maintaining epithelial-mesenchymal cell interactions [2]. In the cancer context, in contrast, fibroblasts may favor the development of age-related proliferative disorders by altering the tissue microenvironment, enhancing several extrinsic tumor-promoting processes and becoming the so-called cancer-associated fibroblasts (CAFs) [3].

Little is known about the molecular mechanisms that lead to activation of CAFs from tissue-resident fibroblasts, circulating marrow-derived fibroblast progenitors or mesenchymal stem cells [2]. Phenotypically, CAFs resemble myofibroblasts, as they are characterized by the expression of α–smooth muscle actin (α-SMA), contractile stress fibers, up-regulated synthesis of ECM and ECM remodeling proteases, resulting in deposition of a reactive stroma. For this reason TGFβ, which is known to be involved in myofibroblast differentiation during wound repair [4], has been long considered the most prominent cancer cell-derived factor able to transform resident fibroblasts into CAFs.

A reactive stroma exhibiting ECM alterations typical of wound healing has been observed in prostate cancer (PCa) and even in precancerous lesions (e.g. PIN), suggesting a role for TGFβ [5]. Accordingly, *in vitro* studies have shown that tumor-derived TGFβ is able to induce activation of human prostate stroma through heavy deregulation of key signalling pathways crucially involved in maintaining tumor-promoting features, including FGF2, CTGF, SDF1, WNT3A and IGF axes [6, 7]. However, mounting evidence demonstrates that CAFs may be a heterogeneous cell population within a single tumor or adopt different phenotypes depending on the tumor type [8]. For example, Planche [9] showed that invasive breast and prostate reactive stromas display incomplete overlap of global gene expression profiles. Moreover, they found a correlation between patient clinical outcome and breast or prostate deregulated stromal genes, but not a common survival predictive gene signature of activated stroma for both tumor types [9].

Different CAF transcriptomic phenotypes may be reflective of activation by different tumor-derived stimuli. In this regard, we have recently showed, in the PCa setting, that tumor-derived interleukin-6 (IL6), via the secretion of soluble factors including metalloproteases, may itself activate normal fibroblasts and subsequently (i) induce epithelial-mesenchymal transition (EMT) in PCa cells, thus increasing their invasive capability, (ii) favor the expression of stemness markers, and (iii) support PCa growth and metastasis *in vivo*, as also observed for patient-derived CAFs [10]. Altogether, such preliminary evidence would suggest a role for IL6 in activating human prostate fibroblasts (HPFs) to CAFs.

In the present study, patient-derived CAFs and HPFs activated *in vitro* with either TGFβ or IL6 were comparatively analyzed for gene and microRNA (miRNA) expression profiles, with the aim to define transcriptional pathways responsible for fibroblast activation and establish whether different subpopulations of CAFs may exist in PCa.

## RESULTS

### Comparative gene expression profiling reveals major transcriptome similarities between IL6-activated fibroblasts and patient-derived prostate CAFs

**Figure 1:**
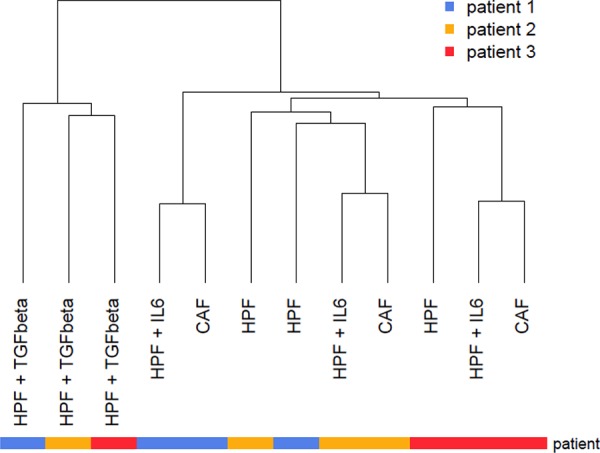
Unsupervised analysis of prostate fibroblast gene expression profiles Gene expression profiles of CAFs, adjacent normal fibroblasts (HPF) and either TGFβ- or IL6-activated fibroblasts were subjected to hierarchical clustering using Euclidean distance and average linkage. Resulting dendrogram was reported and patient ID was color-coded according to the legend.

To analyze the transcriptomic changes associated with fibroblast activation and acquisition of tumor-promoting features, gene expression profiles were comparatively evaluated in fibroblasts derived either from the tumor (Cancer Associated Fibroblasts, CAFs) or from the adjacent non-neoplastic areas (Human Prostate Fibroblasts, HPFs) of three radical prostatectomies (Gleason score 4+5, pT3a, N0). HPFs activated *in vitro* with TGFβ or IL6 have been included in the analysis to understand whether either signals may be able to induce part of the transcriptomic changes occurring in patient-derived CAFs. Unsupervised hierarchical clustering of the specimens revealed that TGFβ-stimulated HPFs were characterized by a markedly different gene expression profile compared to the other fibroblast types (Figure 1). On the contrary, IL6-stimulated HPFs showed transcriptional profiles highly similar to CAFs, driving a “per patient” clustering (Figure 1).

**Figure 2:**
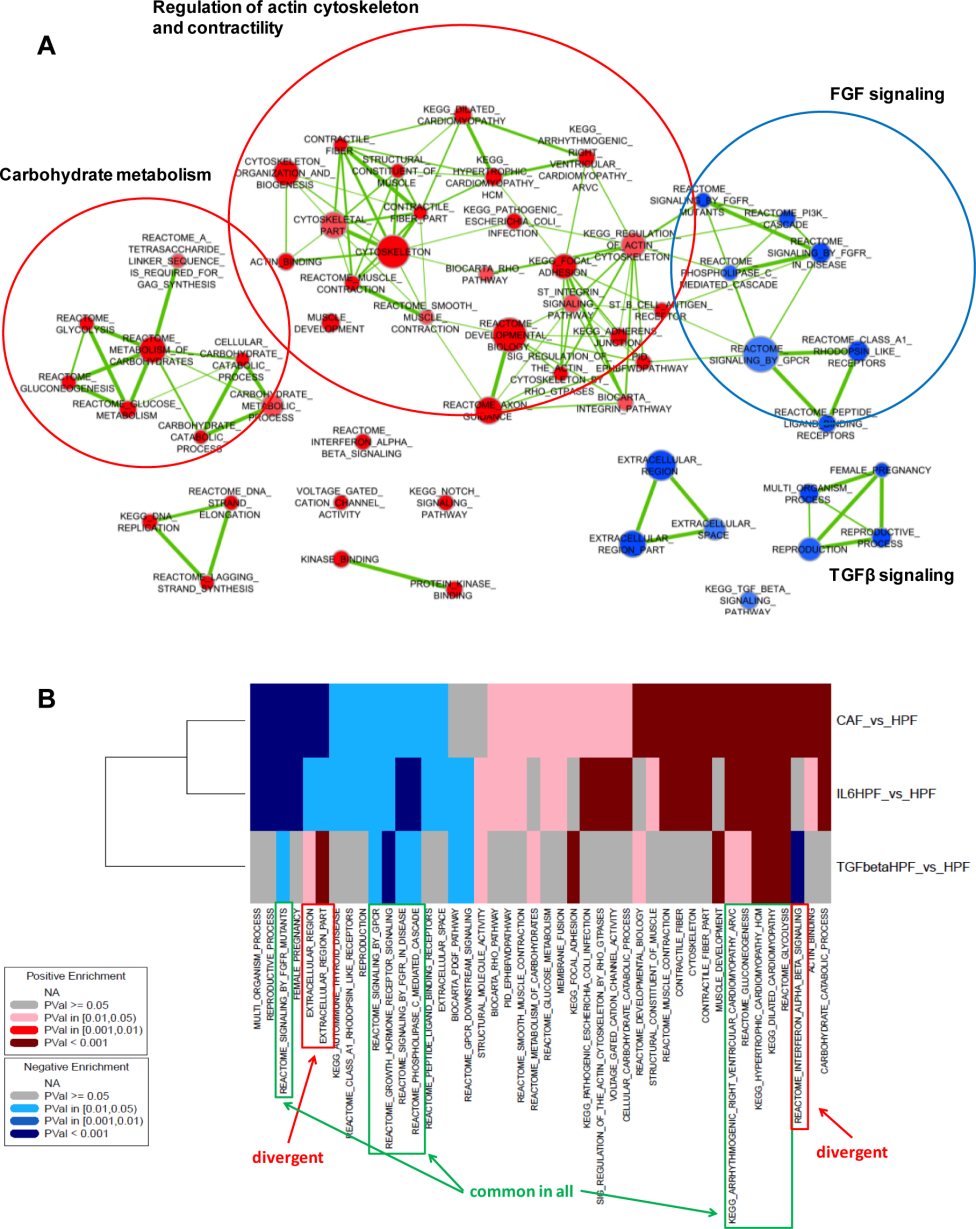
Gene set enrichment analysis **A.** Graphical representation of positively (*red*) or negatively (*blue*) enriched gene sets in CAFs (*n* = 3) compared with HPFs (*n* = 3). Node size is proportional to the number of genes included in the gene set, whereas link thickness is proportional to the number of common genes. **B.** Heatmap summarizing significantly enriched gene sets in at least two of the evaluated comparisons (CAF vs HPF, IL6-HPF vs HPF, TGFβ-HPF vs HPF, *n* = 3 for each group).

To obtain further information about the molecular events occurring in activated fibroblasts, each type of activated fibroblasts was compared to HPFs using a gene set enrichment analysis (GSEA). Initially, we attempted to characterize the transcriptomic phenotype of patient-derived CAFs (Figure 2A). We found positive enrichment of terms related to the actin cytoskeleton remodeling and muscle contractility, thus supporting the well-known similarity between CAFs and myofibroblasts. Intersection of leading edge genes from the enriched gene sets of the cytoskeleton-muscle contraction network revealed major up-regulation of myosin light chain subunits and regulatory proteins, as well as regulators of actin cytoskeleton assembly (Supplementary Table S1). In addition, and in trend with our previous observations [11], CAFs also exhibited enrichment of genes involved in carbohydrate metabolism (Figure 2A). Interestingly, among positively enriched gene sets there were also the “voltage gated cation channel activity” gene set (Supplementary Figure S1), containing genes that code for calcium and potassium channels probably involved in the acquisition of a contractile phenotype, and the “interferon-alpha-beta signalling” gene set, which contains genes that are likely modulated upon cytokine stimulation (including IL6, i.e. Interferon-β) *in vivo* (Figure 2A).

Among down-regulated genes, enrichment was found for terms related to growth hormone/FGF signaling and, surprisingly, TGFβ signaling (including genes coding for SMAD and BMP proteins) (Figure 2A, Supplementary Figure S1). In addition, a “pregnancy–related” gene set was found, mainly consisting of PSG (pregnancy specific beta-1-glycoprotein) glycoproteins, the role of which in cancer stroma is still to be explored (Figure 2A, Supplementary Figure S1).

**Figure 3:**
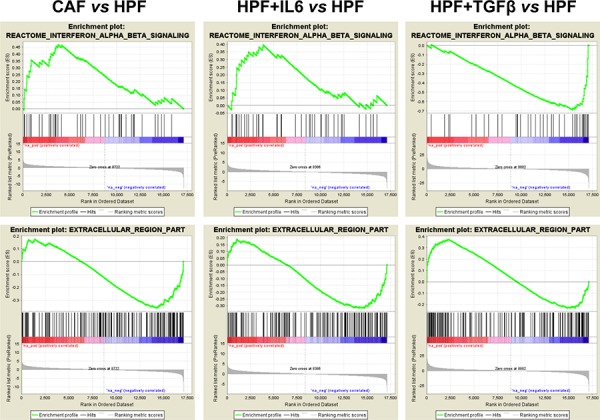
Selected gene set enrichment plots Example of gene sets having divergent enrichment between CAF/IL6-HPFs and TGFβ-HPFs (vs HPF). The analysis was performed using GSEA that calculate an Enrichment Score (ES) by walking down the list of genes ranked according with the t-statistics value of the reported comparison, increasing a running-sum statistic when a gene is in the gene set and decreasing it when it is not (green line). A positive ES indicates gene set enrichment at the top of the ranked list (red part of the horizontal bar); a negative ES indicates gene set enrichment at the bottom of the ranked list (blue part of the horizontal bar). The middle portion of the plot shows where the members of the gene set appear in the ranked list of genes. The bottom portion of the plot shows the value of the ranking metric as you move down the list of ranked genes.

GSEA analysis was then extended to *in vitro* activated fibroblasts for comparative purposes. The positively and negatively enriched terms (*P*-value < 0.05) in at least two comparisons are shown in (Figure 2B). Only few gene sets were commonly enriched in all comparisons: glycolysis/gluconeogenesis and cardiomyopathy–the latter including genes involved in muscle contraction–among positively enriched gene sets, and growth hormone/FGF signaling among negatively enriched terms (Figure 2B, Supplementary Figure S2). In contrast, the gene set “interferon-alpha-beta signalling” was coherently enriched in genes up-regulated in either CAFs or IL6-activated fibroblasts whereas showed an opposite trend in TGFβ-HPFs (Figure 3). Also terms related to extracellular region part showed differential enrichment in CAFs or IL6-HPFs compared to TGFβ-HPFs (Figure 3), corroborating the hypothesis that, despite phenotypic similarities, gene expression of myofibroblasts diverges from that of patient-derived CAFs. Major overlap was indeed observed in gene sets enriched in IL6-stimulated HPFs and CAFs compared with HPFs, including pathways related to contractile fibers, muscle contraction, voltage gated channels (positive enrichment), reproductive process and pregnancy (negative enrichment) (Figure 2B).

**Figure 4:**
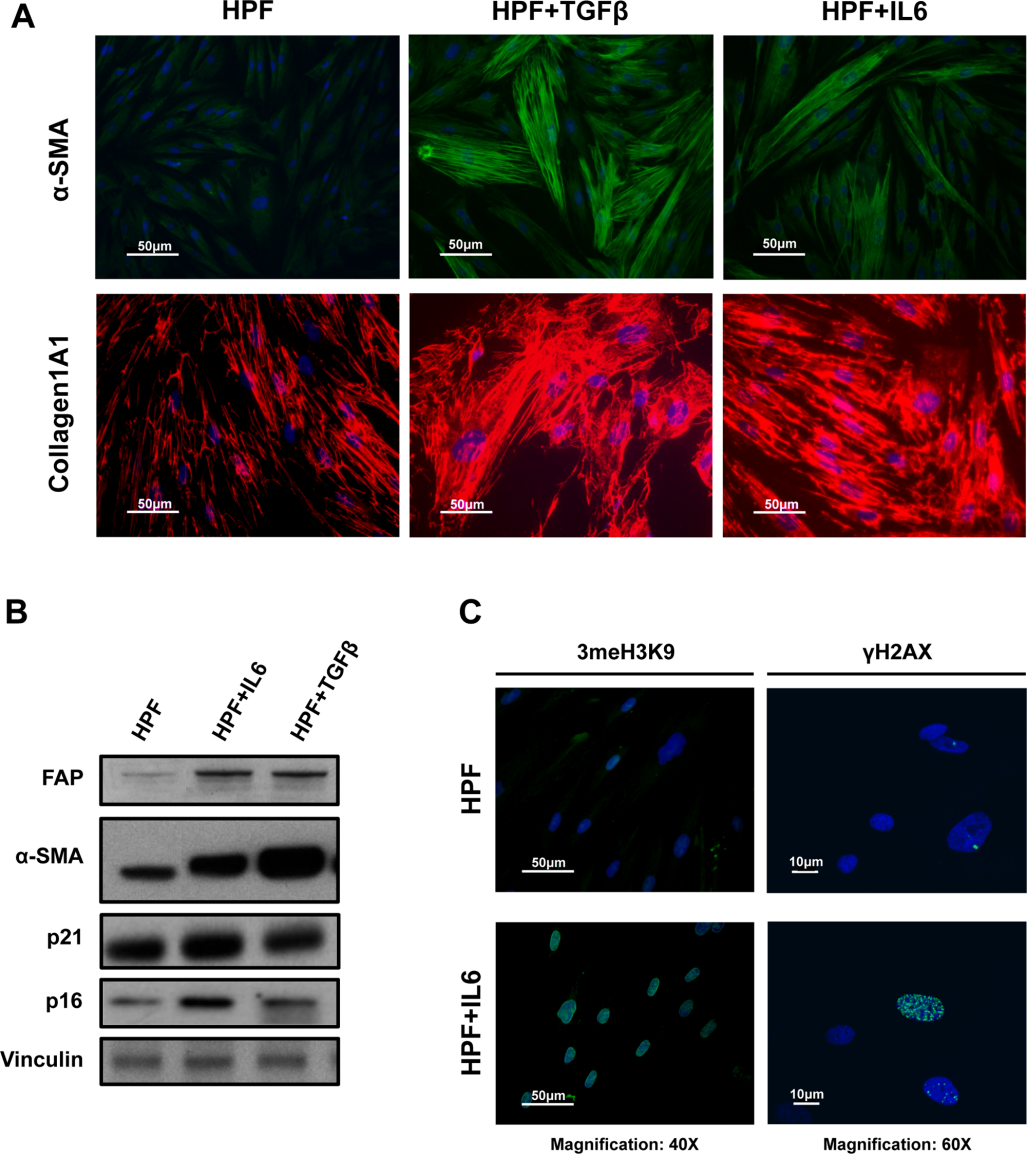
IL6-induced phenoconversion of normal prostate fibroblasts into activated fibroblasts **A.** Representative immunofluorescence images of primary normal prostate fibroblasts (HPFs) treated with IL6 or TGFβ, the latter used as positive control for myofibroblast-like differentiation (Magnification: 40×). α-SMA and Collagen1A1 were stained in green and red respectively. IL6-activated fibroblasts showed increased staining for both markers, suggesting an activated phenotype, though maintaining a characteristic spindle-like shape with extended cellular processes compared to TGFβ-HPFs. **B.** Representative immunoblotting for activation and senescence markers. Fibroblast activation of both IL6- and TGFβ-stimulated HPFs was confirmed by increased α-SMA and FAP protein amounts. IL6-HPFs also showed enhanced expression of senescence-related p21 and p16 proteins. **C.** Representative images of 3meH3K9 (magnification: 40×) and γH2AX (magnification: 60×) staining confirmed senescent-like phenotype of IL6-activated fibroblasts.

Interesting observations emerged by looking at single gene differential expression as well. For example, all activated fibroblasts showed up-modulation of *ACTA2* (i.e., α-SMA), which is consistent with their activated state (Supplementary Table S2). Accordingly, IL6-stimulated HPFs exhibited a more elongated cell morphology when compared to HPFs and were characterized by increased expression of α-SMA (*green*), which was organized into contractile fibers (Figure 4A), similarly to TGFβ-HPFs. Enhanced staining for collagen 1A1 (Figure 4A) and increased fibroblast activation protein (FAP) levels (Figure 4B) were also observed, as assessed by immunofluorescence and immunoblotting, respectively. It is worth mentioning, however, that the most evident up-regulation of α-SMA was observed upon TGFβ stimulation (Figure 4B). TGFβ-stimulated fibroblasts were actually characterized by an enlarged, flatted morphology with dense contractile fibers, whereas IL6-HPFs maintained the fibroblast characteristic fusiform shape with extended cellular processes (Figure 4A). IL6-activated fibroblasts also showed increased levels of p16 and p21 cell cycle inhibitors, which may account for a senescent-like phenotype (Figure 4B). Consistent with this, increased trimethylation of H3 histone on lysine 9 (3meH3K9) and number of γ-H2AX foci (Figure 4C) were detected in HPFs upon IL6 stimulation, confirming our previous evidence showing that activated fibroblasts share features of senescent fibroblasts and viceversa [12].

Differential analysis also highlighted that whilst TGFβ-HPFs were characterized by higher TGFβ1/2 expression levels than normal fibroblasts, CAFs and IL6-stimulated HPFs shared increased expression of the alternative ligand TGFβ3 (Supplementary Table S2). Notably, *TGFB3* mRNA was markedly down-regulated in TGFβ-HPFs, thus suggesting a differential involvement of TGFβ family members and downstream signalling in the different types of activated fibroblasts. Involvement of TGFβ3 pathway in prostatic CAFs has been already reported by van der Heul-Nieuwenhuijsen [13], who also showed opposite regulatory effects of *TGFB3* compared to *FOXF2* transcription factor [13]. Curiously, *PSG* genes, found to be down-regulated only in CAFs and IL6-stimulated HPFs (Supplementary Table S2), are regulated by *FOXF2* [13]. Up-modulation of distinct sets of metalloproteases was also observed in IL6- vs TGFβ-stimulated fibroblasts, namely MMP11 in the former and MMP1-2-10-14-23A in the latter (Supplementary Table S2).

**Figure 5:**
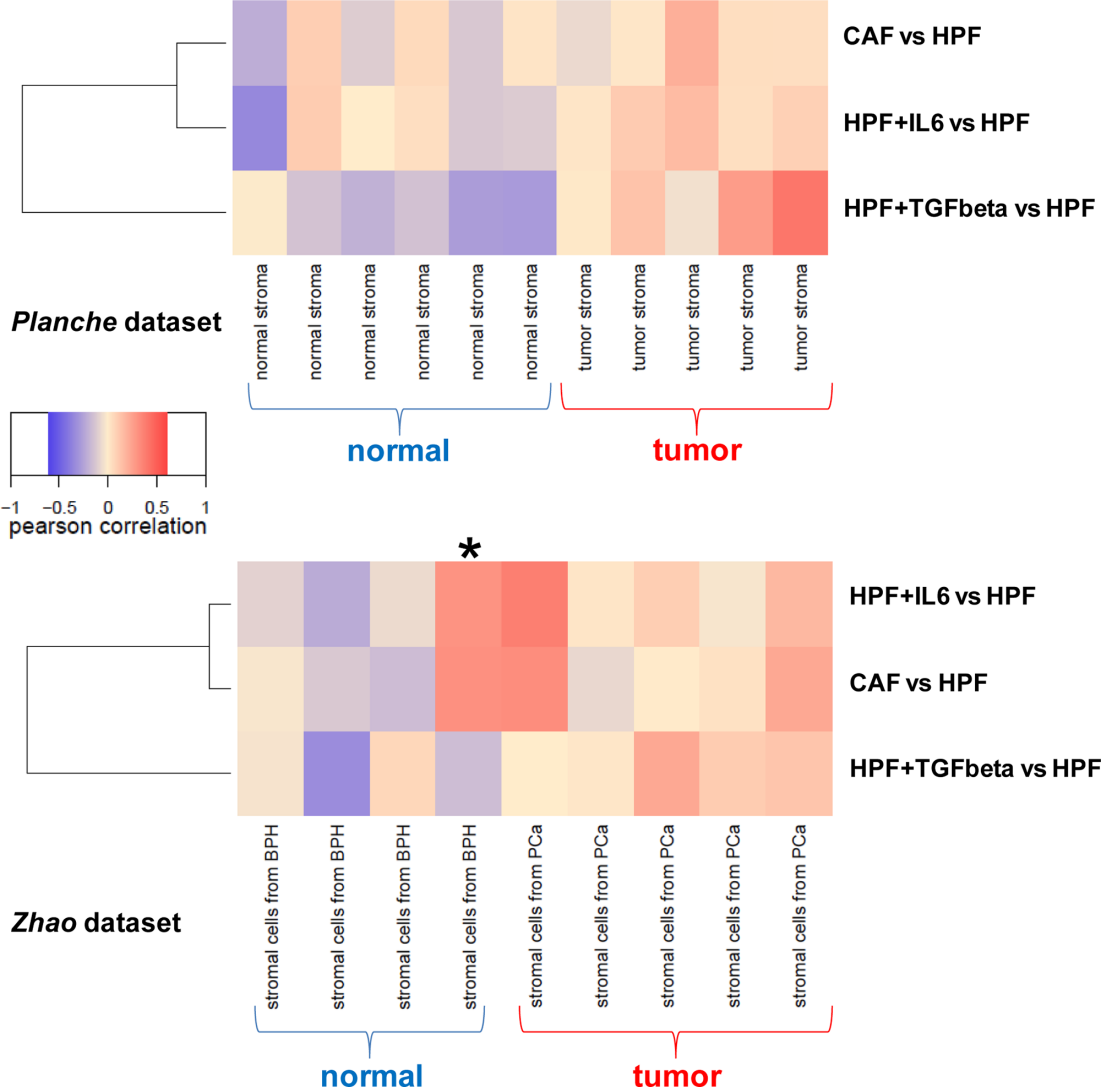
Correlation between activated fibroblast signatures and prostate stroma expression profiles Three distinct centroids were derived from differentially expressed genes between CAF vs HPF, IL6-HPF vs HPF or TGFβ-HPF vs HPF (*n* = 3 for each group), and correlated with expression profiles from microdissected tumor/normal stroma (Planche dataset, *upper panel*) or tumor/normal fibroblasts (Zhao dataset, *lower panel*). Results were summarized as a heatmap, positive correlations are in red and negative correlations in blue.

As next step, gene expression profiles emerging from our analyses have been challenged on publicly available gene expression profiles of prostate microdissected stroma [9] or CAFs [14]. Results showed that, in general, gene expression of tumor stroma was positively correlated with all signatures of activated fibroblasts (CAF, IL6 or TGFβ) (Figure 5). Specifically, though some tumor stroma samples were characterized by a clear TGFβ signature, others rather showed an IL6 profile (Figure 5), thus validating our findings concerning a possible role of IL6 in activating prostate fibroblasts *in vivo*. These findings may suggest the possible co-existence of TGFβ- and IL6-activated fibroblasts within prostate tumor stroma; further investigation will be carried out to understand when and where activation of resident fibroblasts by cancer cells is preferentially driven by TGFβ or IL6. Surprisingly, fibroblasts obtained from a benign prostatic hyperplasia (BPH) specimen in Zhao dataset (marked with an asterisk in Figure 5) were characterized by IL6 and CAF signatures, suggesting that gene expression characteristic of an activated stroma may be found even in lesions that still appear as non-malignant from a histological point of view.

### Comparative miRNA expression profiling identifies miRNAs involved in fibroblast activation induced by different stimuli

**Figure 6:**
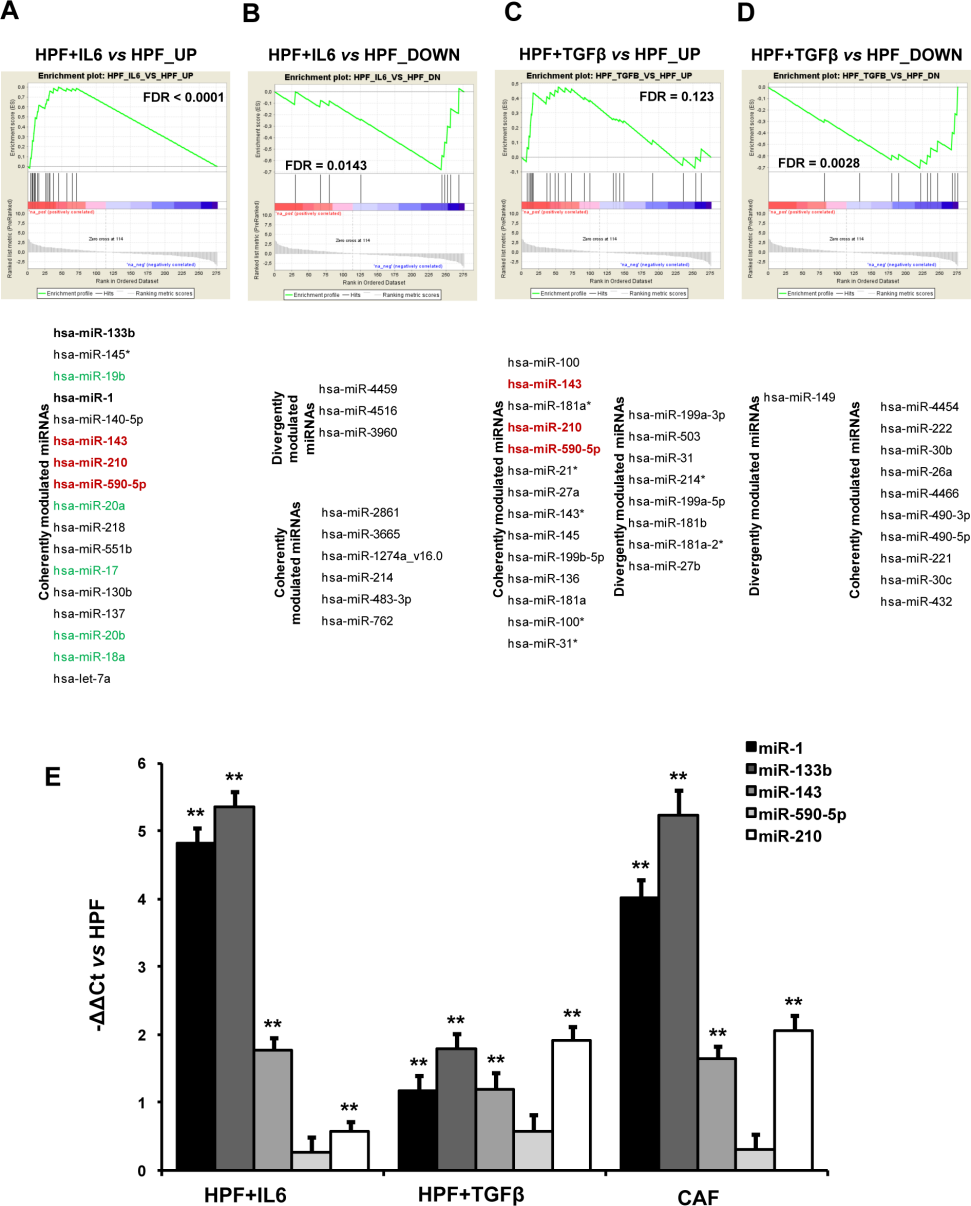
miRNA expression analysis **A–D.** Significantly up- (A) and down-regulated (B) miRNAs in IL6-HPFs (*n* = 3) or up- (C) and down-regulated (D) in TGFβ-HPFs (*n* = 3) compared with HPFs (*n* = 3) were used as miRNA sets tested for enrichment in the comparison between CAF and HPF miRNA expression profiles. Coherent or divergent expression of miRNAs within miRNA sets with respect to CAF/HPF trend is reported. MiRNAs coherently modulated in all types of activated fibroblasts are highlighted in red. In green are miRNAs belonging to miR-17~92 cluster, in bold muscle-specific miRNAs, both groups being coherently modulated in IL6-activated fibroblasts and patient-derived CAFs. **E.** qRT-PCR validation of miRNAs found to be up-regulated in patient-derived CAFs, IL6-HPFs and TGFβ-HPFs. Date were reported as -ΔΔCt with respect to serum-starved HPFs of three independent experiments. **P < 0.01.

MiRNA expression profiling was carried out on the same samples used for gene expression analyses. After quality control, CAF_1 sample, classified as outlier because highly divergent from all the other samples in an unsupervised analysis, was removed and the remaining samples were used to perform a paired class comparison between activated and normal HPFs, as previously described for gene expression analyses. The different sample size would have made a direct comparison between the number of differentially expressed miRNAs biased. As an alternative, the differentially expressed miRNAs (*P*-value < 0.05) in either IL-6 or TGFβ-stimulated fibroblasts vs HPFs were evaluated as a “miRNA set” on the miRNA list ranked according to *t*-statistic values obtained by comparing CAFs vs HPFs. Again we found that both up- and down-regulated miRNAs in IL6-HPFs showed the same trend in CAFs (Figure 6A, 6B). Conversely, miRNAs up-regulated in TGFβ-HPFs were not significantly enriched (FDR = 0.123) in CAFs (Figure 6C), whereas miRNAs repressed in TGFβ–HPFs were also down-regulated in CAFs (Figure 6D). Notably, miRNAs down-regulated in either IL6- or TGFβ-HPFs and commonly repressed in CAFs were specific for each of the two stimuli. For example, CAFs and IL6-HPFs shared the down-modulation of miR-762 (Figure 6B), which is predicted to target α-SMA in its 3′UTR (predictions made by RNA22 tool, [15]). MiRNAs commonly repressed in CAFs and TGFβ-HPFs were instead miR-26a, miR-221/222, miR-490-5p/3p and others (Figure 6D).

**Figure 7:**
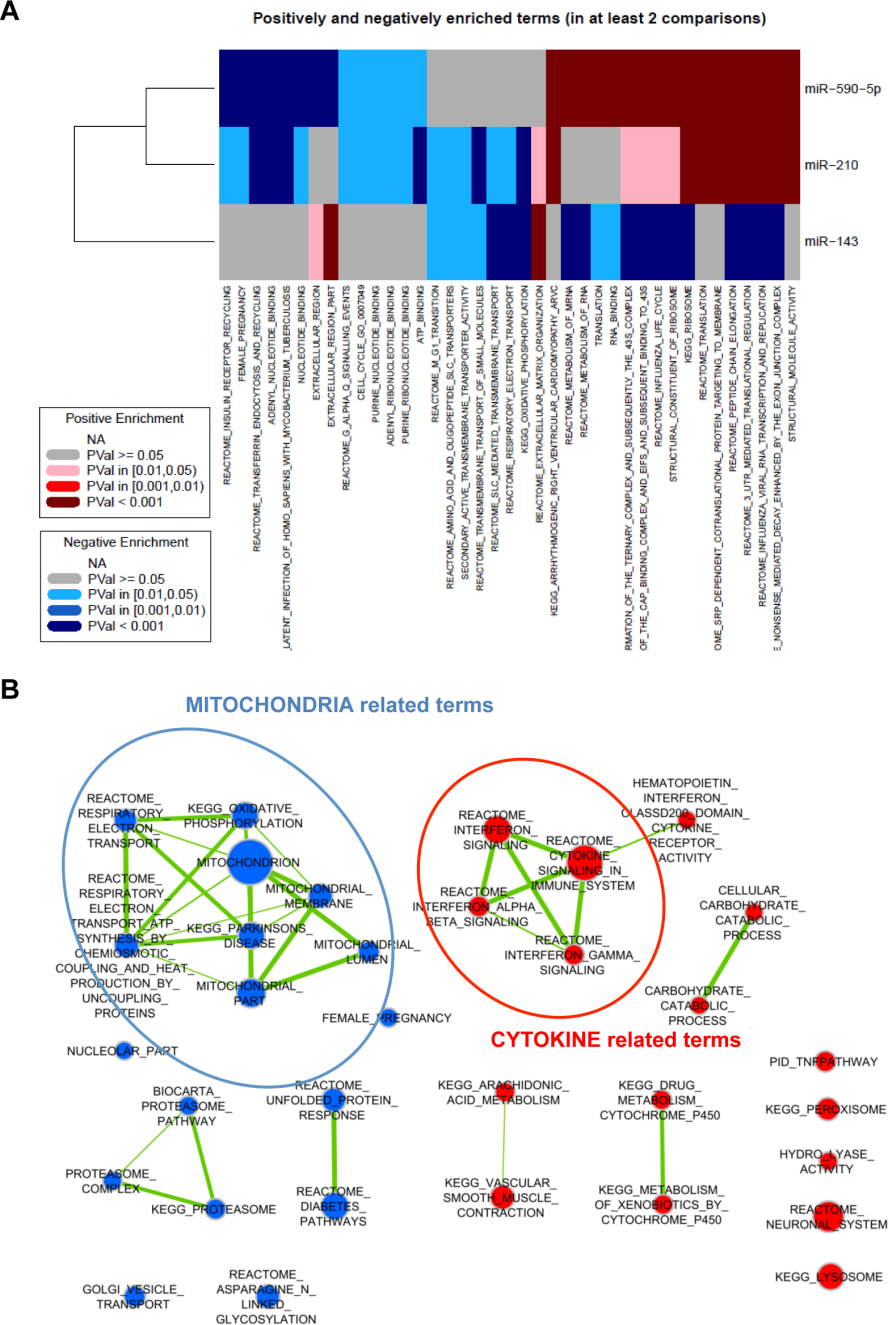
Integration of gene and miRNA expression data **A.** Gene expression data were correlated with expression levels of miR-590-5p, miR-210 or miR-143 respectively. Genes were then ranked according with the correlation value and subjected to gene set enrichment analysis. Enriched gene sets are summarized as a heatmap. Red indicates positive enrichment and blue negative enrichment; darker colors indicate higher statistical significance according to the legend. **B.** The same analysis in (A) was repeated for miR-133b and here represented as a graph. Node size is proportional to the number of genes included in the gene set, whereas link thickness is proportional to the number of common genes. Positive enrichment is in red and negative enrichment in blue.

MiRNAs up-regulated in all activated fibroblasts were miR-210, miR-143 and miR-590-5p (Figure 6A, 6C), a result that was validated by qRT-PCR (Figure 6E). In this regard, we already showed that miR-210 is indeed able to activate HPFs, when ectopically overexpressed [12]. Analysis of genes correlated with each of these miRNAs revealed major commonalities between miR-210 and miR-590-5p, thus suggesting that these two miRNAs may govern the same pathways, such as the regulation of translation (as evidenced by various gene sets among the positively correlated genes) or insulin receptor recycling (among negatively correlated genes) (Figure 7A). Curiously, a negative correlation was found with the term “female pregnancy”, which includes genes that we actually observed to be down-regulated in CAFs. Genes correlated with miR-143 were instead mostly related to ECM organization (positive correlation) or oxidative phosphorylation (negative correlation), both aspects being consistent with the phenotype of all activated fibroblasts (Figure 7A).

IL6-activated fibroblasts additionally shared with patient-derived CAFs up-regulation of miR-17~92 family members, as well as that of muscle- and heart-specific miR-1 and miR-133b (Figure 6A). When tested by qRT-PCR, a marked up-regulation of miR-1 and miR-133b was actually found in IL6-HPFs and CAFs, but even to a lesser extent, up-modulation of the miRNAs was also detected in TGFβ-HPFs (Figure 6E). These two miRNAs are well known as essential rulers of muscle cell function and cardiomyocyte differentiation [16], which is consistent with the enrichment of terms related to muscle contractility and heart morphogenesis found in CAFs (Figure 2A).

### Prototypic IL6-related miR-133b is released by CAFs and induces *per se* fibroblast activation

**Figure 8:**
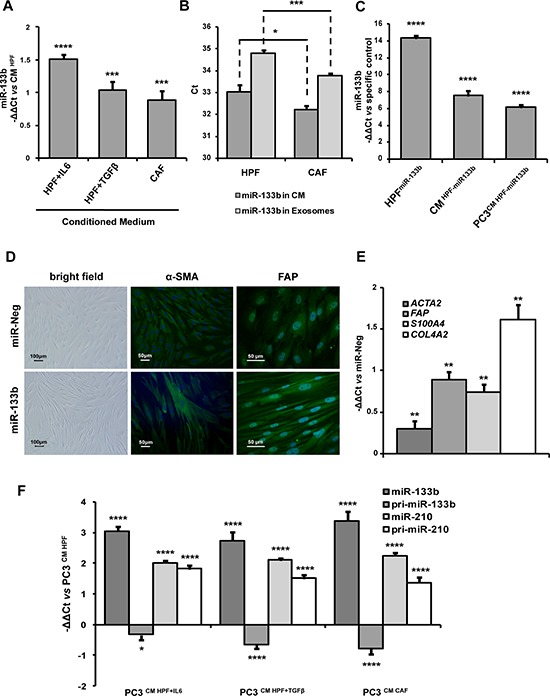
miR-133b induces fibroblast activation and acts as soluble factor for paracrine stimulation of fibroblast and tumor cells **A.** miR-133b expression levels were evaluated in conditioned medium (CM) from CAFs, IL6- or TGFβ-activated fibroblasts by qRT-PCR. miR-133b release in CM increased upon stimulation of HPFs with IL6 and, to a lesser extent, with TGFβ. Enhanced secretion of miR-133b was also found in CAFs. Data were analyzed using spike-in non-human synthetic miRNA as normalization control and were reported as −ΔΔCt with respect to CM from serum-starved HPFs of three independent experiments. **B.** miR-133b expression levels were evaluated in CM and extracellular exosomes from CAFs and HPFs by qRT-PCR. CAFs showed enhanced release of both total and exosome-associated miR-133b compared to HPFs. However, starting from equal RNA amounts, Cts were higher in exosomes (hence miR-133b expression lower) with respect to total media, suggesting that miR-133b may be secreted in forms other than exosomes. **C.** qRT-PCR measurement of endogenous miR-133b levels in HPFs transfected with miR-133b mimics (vs cells transfected with miR-Neg), of miR-133b in the media of HPFs transfected with miR-133b mimics (vs media form cells transfected with miR-Neg), and of endogenous miR-133b in PC3 cells stimulated with CM from miR-133b-transfected cells (vs cells stimulated with CM from miR-Neg-transfected HPFs). Data were reported as −ΔΔCt respect to specific control of three independent experiments. **D.** Representative bright field and immunofluorescence (α-SMA and FAP staining) images of HPFs transfected with miR-133b mimics or miR-Neg (Magnification: 10×). Ectopic expression of miR-133b induced morphological changes and increase of activation markers reminiscent to those observed in IL6-activated fibroblasts. **E.** qRT-PCR assessment of ACTA2, FAP, S100A4 and COL4A2 to confirm fibroblast activation upon miR-133b overexpression in HPFs. qRT-PCR data were reported as −ΔΔCt respect to miR-Neg-transfected HPFs of three independent experiments. **F.** pri-miR-133b, miR-133b, pri-miR-210 and miR-210 expression levels were assessed in PC3 cells upon stimulation with CM from HPF-IL6, HPF-TGFβ or from CAFs by qRT-PCR. Results showed direct uptake of miR-133b in PC3 cells from fibroblast media, as shown by paradoxical down-regulation of endogenous pri-miR-133. In contrast, fibroblast stimulation increased miR-210 expression in PC3 cells by enhancing transcription of pri-miR-210, suggesting a different mechanism. Data were reported as −ΔΔCt with respect to PC3 cells stimulated with CM from HPFs. *P < 0.05; **P < 0.01; ***P < 0.001; ****P < 0.0001.

MiR-133b was chosen as a prototype of miRNAs up-regulated in IL6-stimulated fibroblasts, as it was also the top ranking in CAF vs HPF comparison (Figure 6A). Analysis of genes correlated with miR-133b revealed negative correlation with mitochondrion-related genes, including those involved in oxidative phosphorylation and respiratory chain (Figure 7B), with this finding being in trend with the glycolytic metabolism of CAFs. Among positively correlated genes, enrichment for terms related to cytokines and interferon signalling was found (Figure 7B). This may be related both to miR-133b up-regulation found in CAFs as a consequence of IL6 (and possibly other cytokines) stimulation, as well as to a role for the miRNA in regulating the secretion of soluble factors. In this regard, it was striking to find that the levels of miR-133b were also increased in the conditioned media of primary normal fibroblasts activated *in vitro* with IL6 or TGFβ as well as those of patient-derived CAFs compared to the media obtained from non-activated HPFs, with a major release found upon IL6 stimulation (Figure 8A). Possibly, miR-133b may itself represent a soluble factor secreted by activated fibroblasts to support paracrine activation of non-activated fibroblasts or promote tumor progression. When exosomes were isolated from the fibroblast media, again we found increased abundance of miR-133b in exosomes from CAFs compared to those from HPFs (Figure 8B). However, comparison of miR-133b threshold cycles between whole media and exosomal fraction preliminarily suggested that miR-133b may be only in part released through exosomes (Figure 8B).

To investigate miR-133b ability to induce fibroblast activation, primary normal fibroblasts were directly transfected with miR-133b. Overexpression of the miRNA (as assessed by qRT-PCR, Figure 8C, left bar) resulted in marked morphological changes, with increase in the number of elongated cells reminiscent of IL6-stimulated fibroblasts (Figure 8D). Accordingly, qRT-PCR revealed up-modulation of a number of fibroblast activation markers, such as *ACTA2*, *FAP*, *S100A4* and *COL4A2* (Figure 8E). In addition, immunofluorescence analysis confirmed changes in morphology together with increased staining for α-SMA and FAP (Figure 8D). Overexpression of miR-133b resulted in an enhanced secretion of the miRNA, as assessed by qRT-PCR on the media of transfected cells (Figure 8C, middle bar), thus confirming miR-133b as one of the mediators of the secretory phenotype of activated fibroblasts. Strikingly, endogenous miR-133b expression levels also increased in PC3 cells stimulated with the conditioned medium of miR-133b-activated fibroblasts (Figure 8C, right bar). Even to a lesser extent, miR-133b levels also increased in PC3 cells stimulated with media from IL6- or TGFβ-activated fibroblasts, or CAFs (Figure 8F). As the levels of miR-133b primary transcript were even down-regulated in stimulated PC3 cells (Figure 8F), it is conceivable that tumor cells may uptake mature miR-133b directly from the medium of activated fibroblasts and, as a consequence, repress the endogenous transcription of the miRNA.

A different scenario emerged for miR-210, up-regulated in all types of activated fibroblasts (Figure 6A, 6C) and able *per se* to activate HPFs when ectopically overexpressed [12]. In fact, the expression levels of both the mature and the primary transcript of miR-210 increased in PC3 cells upon stimulation with activated fibroblasts, thus suggesting a direct transcriptional activation rather than a transfer from the fibroblast media, as instead observed for miR-133b (Figure 8F). In this regard, we have previously shown that CAFs elicit in PCa cells a pro-oxidant response, which culminates in the overexpression of hypoxia-inducible factor-1 (HIF-1), which in turn regulates transcription of its targets, including miR-210 [10, 17, 18].

**Figure 9:**
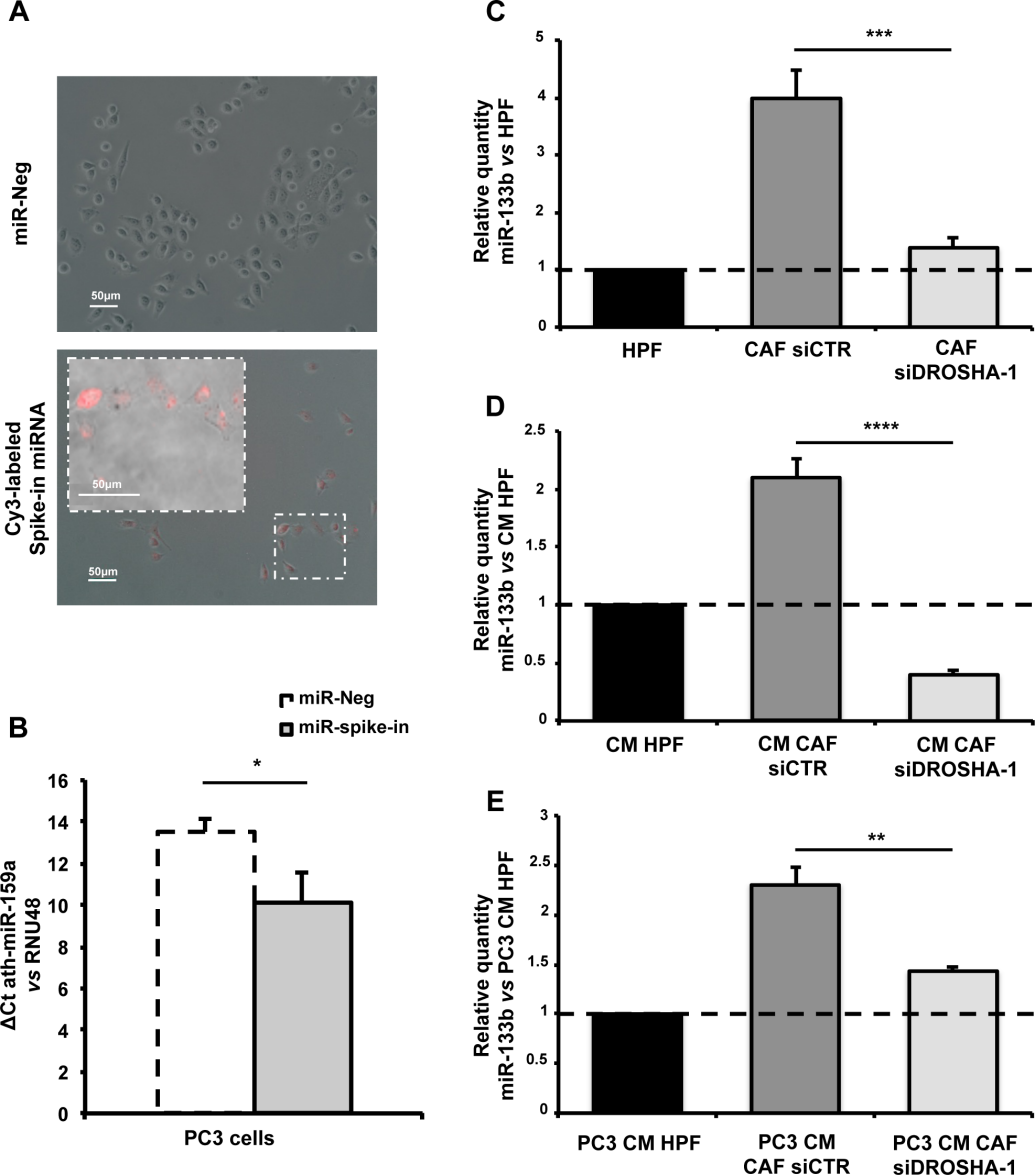
PC3 cells directly internalize miR-133b from fibroblast media **A.** Merged bright field and fluorescence images of PC3 cells grown in a medium supplied with Cy3-labeled spike-in miRNA (Cy3™ Dye-Labeled Pre-miR Negative Control #1, Life Technologies) or miR-Neg as a control (Magnification: 20×, insert 40×). **B.** qRT-PCR showing ath-miR-159a expression levels in PC3 cells were grown in a medium supplied with ath-miR-159a spike-in miRNA or miR-Neg as a control. Data are presented as ΔCt compared to RNU48. Notably, signal for ath-miR-159a in miR-Neg sample was undetermined and Ct set to 40. **C.** Expression levels of endogenous miR-133b in CAFs transfected with siCTR or siDROSHA-1. Data are presented as relative quantity vs HPFs. **D.** Expression levels of miR-133b in the media from CAFs transfected with siCTR or siDROSHA-1. Data are presented as relative quantity vs CM from HPFs. **E.** Expression levels of miR-133b in PC3 cells stimulated with the media from CAFs transfected with siCTR or siDROSHA-1. Data are presented as relative quantity vs PC3 cells stimulated with CM from HPFs. *P < 0.05; **P < 0.01; ***P < 0.001; ****P < 0.0001.

To confirm the capability of PC3 cells to directly internalize miRNAs present in fibroblast media (i.e. miR-133b), we performed a couple of experiments where a spike-in miRNA was added to the medium prior stimulation of PC3 cells. In the first setting, a Cy3-labeled miRNA mimic was used and miRNA internalization was assessed by fuorescence microscopy. In the latter, we used a non human miRNA (ath-miR-159a), and miRNA uptake by tumor cells was evaluated by qRT-PCR for ath-miR-159a. In both settings, after a 24 h-stimulation, PC3 cells were washed thrice to remove any residual miRNA remaining on cell surface. Results showed that PC3 cells can readily internalize the miRNA present in the medium, as evidenced by fuorescent signal within the cytoplasm of cells stimulated with labeled miRNA (Figure 9A). In addition, PCR signal for non human ath-miR-159a became detectable in cells grown in the presence of synthetic ath-miR-159a (Figure 9B), suggesting direct uptake from the medium. To ultimately confirm that miR-133b expression levels in PC3 cells stimulated by CAFs reflect the amount of miR-133b present in the fibroblast media, we attempted to modulate miR-133b expression in such media and then analyzed miRNA levels in stimulated tumor cells. To this purpose, we silenced *DROSHA*, one of the major components of miRNA processing machinery, in CAFs to reduce miRNA production. We tested two different siRNA molecules and found that siDROSHA-1 was more efficient in repressing *DROSHA* mRNA expression (Supplementary Figure S3). Accordingly, miR-133b endogenous levels decreased in CAFs upon *DROSHA* silencing by siDROSHA-1 (Figure 9C), lowering to levels found in HPFs (Figure 9C). Coherently, miR-133b levels decreased in the media from siDROSHA-1-transfected CAFs (Figure 9D), which were then used to stimulate PC3 cells. Again, we found that endogenous miR-133b levels in PC3 cells increased upon stimulation with CM from siCTR-transfected CAFs compared to CM from HPFs (Figure 9E). However, *DROSHA* silencing and consequent reduced secretion of miR-133b by siDROSHA-1-transfected CAFs resulted in less efficient uptake of miR-133b by PC3 cells (Figure 9E).

Overall these experiments showed that PC3 cells directly internalize miR-133b from fibroblast media and that endogenous miRNA levels in tumor cells depend on the amount of miR-133b released by fibroblasts.

## DISCUSSION

It is widely accepted that solid tumors are heterogeneous and complex systems consisting of neoplastic cells and untransformed stroma components. Such reactive-stroma is a mixture of immune cells, endothelial cells and CAFs exhibiting activated phenotypes. CAFs are the predominant cell population in PCa microenvironment and are characterized by a phenotype reminiscent to that of fibroblasts involved in wound repair events [5]. Reciprocal interaction between cancer cells and CAFs influences each step of tumor development, growth and metastasis, mainly through the release of soluble growth factors [19]. In the context of PCa, we have previously shown that CAFs, through secretion of MMPs, elicit EMT and achievement of stem cell traits in cancer cells, as well as enhancement of tumor growth and development of spontaneous metastases [10]. We also reported that CAFs induce a Rac1b/cycloxygenase-2-mediated release of reactive oxygen species in carcinoma cells, which activates nuclear factor-κB and HIF-1 [17], and ultimately results in repression of miR-205 transcription. Inhibition of miR-205 function relieves a brake on its target genes, including E-cadherin transcriptional repressors, thus leading to establishment of EMT and enhancement of metastasis [18]. The other way around, tumor cells *per se* contribute to the formation of a reactive stroma. In this regard, we showed that PCa cells can activate normal fibroblasts to a phenotype reminiscent to that of patient-derived CAFs through the secretion of soluble factors, including IL6. In fact, stimulation of HPFs with the medium of castration-resistant PCa cells was sufficient to increase α-SMA and FAP expression and to promote the acquisition of tumor-promoting properties, a phenotype abrogated by the administration of an anti-IL6 antibody [10].

In the present study, we comparatively analyzed gene expression profiles of CAFs and HPFs obtained from radical prostatectomies to contribute to the understanding of the biology and signaling mechanisms involved in reactive stroma formation. Previous studies suggest that, compared to normal fibroblasts, CAFs found in tumors are characterized by enhanced collagen synthesis, secretion of growth factors and ECM modulators, and the activation of unique expression programs [20, 21]. Consistently, we found that CAFs are phenotypically similar to myofibroblasts, with elevated expression levels of α-SMA and positive enrichment of genes that regulate actin cytoskeleton remodeling and muscle contractility. Moreover, activated fibroblasts showed positive enrichment for gene sets related to glycolysis/gluconeogenesis pathway, in accord to our previous evidence that CAFs undergo a metabolic reprogramming from a respiratory to a glycolytic metabolism to support cancer progression [11]. CAFs also exhibited positive enrichment of gene sets related to “voltage gated cation channel activity”, as already observed in microdissected reactive stroma of grade 3 PCa [22]. Among down-regulated gene sets, a “pregnancy-related” signature, mainly composed of PGS glycoproteins, was also found. However, the role of either signaling pathway in supporting the activation or function of PCa stroma is still to be explored.

Very scanty information is currently available on which is the cell population committed to generate CAFs. Lineage-tracing experiments showed that CAFs might evolve from tissue-resident mesenchymal stem cells or, by endothelial-to-mesenchymal transition, from endothelial cells, from vimentin-positive periacinar cells or from vessel-associated pericytes [21, 23, 7]. In addition to these local sources, circulating bone marrow-derived cells known as fibrocytes can be recruited and differentiated into myofibroblasts to support cancer progression. A further possible origin of CAFs is represented by smooth muscle cells. Normal prostate stroma is enriched of this particular cell population, which, during PCa progression, is actually replaced by CAFs [21, 23]. Moreover, several pieces of evidence suggest that CAFs might also derive from fibroblasts resident in the tissue affected by tumor, through direct activation by cancer-derived stimuli [7, 24].

Several boosts, such as cytokines, growth factors, TGFβ and HGF have been shown to regulate reactive stroma formation, by affecting directly or indirectly stroma cells [25]. Among these, TGFβ is the most important and well-studied candidate in maintaining the features of tumor-associated stroma, due to its pivotal role in wound repair and fibroproliferative diseases [4]. *In vitro* studies reported that TGFβ is able to induce normal prostate fibroblasts to acquire a myofibroblast-like reactive phenotype with overexpression of α-SMA protein, components of ECM and enhanced synthesis of collagen type 1– all features well documented in CAFs derived from patients [26, 27]. Paradoxically, however, ablation of TGFβ receptor 2 (TGFBR2) in prostatic fibroblasts resulted in spontaneous neoplasia, illustrating a tumor-suppressive function of TGFβ signaling in early tumor development [28]. It can be hypothesized that, in the PCa context, TGFβ may be more relevant for maintaining CAF phenotype rather that providing stroma with tumor-initiating features. Accordingly, *in vivo* evidence suggests that TGFβ signaling activation in stroma cells has little impact on tumor initiation phase but is critical for all subsequent steps of tumor progression, where maintenance of wound healing-like niche may support tumor aggressiveness [29]. In general, accumulating evidence demonstrates that CAFs may be a heterogeneous cell population within a single tumor or adopt different phenotypes depending on the tumor type, suggesting both inter and intra-tumor stromal heterogeneity [8].

To better understand the signaling mechanisms involved in the formation of PCa-associated stroma and which tumor derived-stimuli are able to induce the reactive phenotype seen *in vivo*, we extended gene expression analysis on fibroblasts activated *in vitro* with TGFβ or IL6. We found that only few gene sets were coherently enriched in both types of activated fibroblasts and superimposable on those found in patient-derived CAFs. In contrast, the “interferon-alpha and beta signaling” and genes related to ECM regulation were positively enriched in both CAFs and IL6-activated fibroblasts but showed an opposite trend in TGFβ-HPFs. Globally, in our hands, a major overlap was found between transcriptomes of IL6-activated HPFs and CAFs compared to TGFβ-HPFs, though both *in vitro* activated fibroblasts showed phenotypic similarities with CAFs. The comparison with publicly available gene expression profiles revealed that, in general, all the three signatures of activated fibroblasts from our study positively correlated with those of PCa microdissected stroma or CAFs, and negatively correlated with normal counterparts. This finding supports the robustness of our data and again confirms the existence of IL6-driven activation patterns in PCa reactive stroma. However, these data also account for the existence of more classical TGFβ-activated myofibroblasts in PCa. It is likely that depending on tumor stage, patient age, cancer etiology (e.g. history of inflammatory diseases, such as prostatitis) or even tumor genetic background, different stimuli may be predominant in inducing activation of a reactive stroma. In this regard, it is worth mentioning that, for example, IL6-activated fibroblasts showed evidence of a senescent-like phenotype (induction of p16, p21, γ-H2AX foci and 3meH3K9), which may account for an age-related activation process. Banerjee [30] already reported that tumor-derived IL6 is able to mediate an epigenetic silencing of DNA damage repair and reactive oxygen-metabolism genes in prostate fibroblasts, ultimately resulting in DNA-damage associated secretory phenotype, characterized by H3 lysine 9 trimethylation and TGFBR2 down-regulation. Notably, reduced TGFBR2 expression levels were also found in our IL6-activated fibroblasts and CAFs (*not shown*). In contrast, TGFβ-dependent activation may be prevalent in conditions that mimic wound healing, such as chronic inflammation.

Gene expression profiling studies of CAFs derived from large cohorts of PCa patients covering all possible sources of stromal heterogeneity are hence warranted to understand the prevalence of IL6- rather than TGFβ-driven CAFs in prostate reactive stroma. In addition, it must be noted that, at present, molecular markers of stroma subpopulations are still poorly defined. Activated fibroblasts are usually identified by their characteristic fusiform-shape, dense cytoskeletal fibres and increased expression of various mesenchymal proteins, such as vimentin, α-SMA and FSP1. However, other markers -not expressed in all fibroblasts- still need to be characterized to identify the multiple populations of fibroblasts and more properly define the tumor-derived stimuli able to trigger activation of specific reactive stroma subtypes.

MiRNA expression profiling confirmed the evidence arisen from gene expression analyses, namely that modulations in the miRnome mediated by IL6 stimulation are the same found in patient-derived CAFs compared to their non-activated counterparts and that only minor overlap exists between these two types of fibroblasts and TGFβ-HPFs. Nevertheless, a small set of miRNAs, including miR-210, miR-143 and miR-590-5p, was found to be coherently up-modulated upon activation induced by either stimulus, suggesting that fundamental processes sustaining reactive stroma formation may be regulated by such miRNAs. In this regard, we already showed that miR-210 is indeed able to convert HPFs into CAF-like cells, promote EMT in PCa cells, support angiogenesis and recruit endothelial precursor cells and monocytes/macrophages [12].

Among miRNAs more specifically (even not exclusively) modulated in IL6-activated stroma was miR-133b. The miRNA belongs to the cluster miR-206/133b, which has been shown to be highly expressed, together with miR-1/133a cluster, in the musculatures of flies, mice and humans and has been largely characterized as involved in regulating muscle cell function [16]. miR-133 is also the first most abundant miRNA in the heart, where it has been shown to regulate genes involved in cardiac contractility, hypertrophy and electric conductance [31]. When administered together with a combination of cardiac transcription factors, miR-1 and miR-133 have been shown to foster conversion of human adult fibroblasts into cardiomyocytes [32].

Here we showed that ectopic overexpression of miR-133b is able *per se* to promote fibroblast activation by inducing phenotypic changes similar to those found in prostate CAFs *in vivo*. An intriguing scenario is hence emerging concerning a possible similarity between prostate CAFs and myocardiocytes, both cell types characterized by enhanced contractility. If properly validated by additional experimental data, this finding may open new opportunities for the translation of drugs already used in the cardiovascular field into anticancer regimens, with the ultimate goal of interrupting the tumor-supportive spur provided by reactive stroma. Potentially, to interfere with myocardiocyte-like phenotype of prostate CAFs, we may envision using antiarrhythmic drugs, which all function by reducing myocardiocyte contractility, though through different mechanisms. Actually, antiarrhythmic drugs have been classified into 4 classes (Vaughan-Williams classification) based on their mechanism of action. For translation into regimens aimed to impair with the functions of tumor reactive stroma, class I (sodium-channel blockers, such as procainamide, mexiletine or flecainide), class III (potassium-channel blockers, such as amiodarone) or class IV (calcium-channel blockers, verapamil) drugs may be tested, based on our evidence that sodium, potassium and calcium voltage-gated channel are highly up-regulated in CAF vs HPFs (Supplementary Figure S1). By mining the literature we found that some of the aforementioned drugs have been used in anticancer schedules to potentiate efficacy of chemotherapy, as is the case of verapamil (a clinical trial described in [33]) or amiodarone (*in vitro* studies [34]) which inhibit the activity of the efflux pumps responsible of multidrug resistance, or as analgesics (mexiletine, flecainide) [35]. Procainamide has been also shown to act as nonnnucleoside inhibitor of DNA methyltransferases in human cancer cells [36].

Our findings further showed that miR-133b is also released into the media of activated fibroblasts and is possibly taken up by PCa cells, where it may contribute to establish a mesenchymal phenotype. This may represent an additional mechanism by which CAFs induce EMT in tumor cells, through the direct transfer of miRNAs typically expressed in cells of the mesenchymal lineage. Moreover, it is likely that released miR-133b may act as a paracrine stimulus able to expand the reactive phenotype to adjacent fibroblasts, thus contributing to extend the stromal niche able to support cancer progression.

miR-133b has been consistently found as down-regulated in prostate carcinomas compared to normal tissues in different studies [37; reviewed also by Gandellini 38]. In our hands however, expression of the miRNA seems to be prevalent in fibroblasts compared to epithelial cells, even if tumor cells up-regulate the miRNA when in contact with CAFs. As a consequence, it is possible that most of miR-133b expression found in normal tissue samples could come from cells of mesenchymal origin, and that down-regulation in tumor specimens found by different authors could mostly reflect a higher epithelial (tumor) vs stromal cell ratio in the sample rather than a direct down-modulation of the miRNA in tumor cells. Actually, no proof has been provided on miR-133b silencing in epithelial cells along tumorigenesis. In addition, miR-133b down-regulation seems to be common to different tumor types [39], a scenario reminding that of miR-143/145 (notably our data suggest that also miR-143 is up-regulated in CAFs). Indeed, down-regulation of such miRNA cluster has been repeatedly reported in different epithelial tumor types (including PCa). However, based on available data on the literature, a possible role for miR-143/145 as cell-autonomous epithelial tumor suppressors is still controversial [40]. Similarly, contrasting results have been obtained regarding the oncogenic or oncosuppressive role of miR-133b in PCa models [37, 41–43]. Such discrepancies may rely on the fact that overexpression of miR-133b in PCa cells generates artificial findings, as the miRNA mainly acts as a master regulator of mesenchymal features in cells of mesenchymal origin (e.g. fibroblasts) or in tumor cells that undergo EMT.

Overall, we provided evidence on how an integrated gene and miRNA profiling may supply interesting hints on pathways relevant for fibroblast activation and maintenance of a reactive stroma, with important implications for the understanding of the mechanisms of cancer progression. In addition, the study allowed us to generate useful working hypotheses for the formulation of novel therapeutic agents based on the targeting of tumor stroma.

## MATERIALS AND METHODS

### Microarray experiments and data processing

Microarray experiments were run by the Functional Genomics Unit/Service from the Fondazione IRCCS Istituto Nazionale Tumori, Milan, Italy. Extracted RNA was processed for gene expression analysis using the Illumina HumanHT-12 v4 chips (47,324 probes) as previously described [44]; Briefly, 800 ng of total RNA were reverse transcribed, labeled with biotin and amplified overnight using the Illumina RNA TotalPrep Amplification kit according to manufacturer's protocol. One μg of the biotinylated cRNA was mixed with the Hyb E1 hybridization buffer and then hybridized at 58°C overnight. Array chips were washed with manufacturer's E1BC solution, stained with 1 μg/mL Cy3-streptavidine and scanned with the Illumina BeadArray Reader. Raw data were generated using the Illumina BeadStudio 3.8 software and processed using the *lumi* package [45] of Bioconductor. After quality control, data were log_2_ transformed and normalized using the Robust Spline Normalization algorithm. Probes with a detection of *P* > 0.01 in all samples were filtered out and, for each gene, the probe with the highest detection rate or higher interquartile range was retained for downstream analyses.

For miRNA expression analysis, SurePrint G3 Human miRNA 8x60K microarrays from Agilent Technologies (custom design based on miRBase 17.0) were used as previously described [46]. Two μg of RNA were labeled and processed according to the manufacturer's recommended protocol. Samples were dephosphorylated with calf intestinal alkaline phosphatase, followed by denaturation in the presence of dimethyl sulfoxide. A dye, cyanine 3-pCp, was then coupled to the dephosphorylated single-stranded RNA by T4 RNA ligase. After hybridization for 20 h at 55°C, the arrays were washed in Agilent GE Wash Buffers, following the manufacturer's instructions. All slides were immediately scanned at 2 mm resolution using an Agilent DNA microarray scanner. The resulting images were analyzed using Agilent's Feature Extraction software v10.7.

One to 4 different probes can target each miRNA and each probe is spotted 10 to 40 times on the array. The total gene signal for each miRNA was obtained by summing the probe signals derived with the Agilent Feature Extraction software. Each probe was defined as detected if its value is greater than 3 times its standard error, and each miRNA was defined as detected if at least one of the probes was detected. Data were log_2_ transformed and normalized using the Robust Spline Normalization algorithm. Only human (“has”) miRNAs detected in at least two samples were selected for downstream analyses. Both gene and miRNA expression data were deposited to the Gene Expression Omnibus data repository (GSE68166) [47].

### Unsupervised and supervised analyses

Hierarchical clustering using Euclidean distance and average linkage was applied for an unsupervised analysis of the data. Class comparison analyses were performed using a linear modeling approach and empirical Bayes methods as implemented in the *limma* Bioconductor package [48]. Gene set enrichment analysis was performed using GSEA v4.0 [49] on the gene list ranked according with the modified *t*-statistics from the class comparison analysis. A gene set collection including canonical pathways and signatures from the literature (C2), as well as gene ontology terms (C5) from the MSigDB database (http://www.broadinstitute.org/gsea/msigdb) were tested for enrichment. The gene sets with *P* < 0.01 were considered significantly enriched and represented as functional enrichment network using a Cytoscape plugin [50].

### Centroid based signature evaluation in publicly available datasets

Differentially expressed genes [*p*-val < 0.01 for CAF or IL6-HPF vs HPF comparison; adj-p-val < 0.0001 for TGFβ-HPF vs HPF (in order to have a similar number of genes)] identified by class comparison analysis for each of our comparisons were used to define three different centroids representative of activated fibroblasts. The expression level of genes included in each centroid was calculated as the average expression in activated fibroblasts (CAF, IL6 and TGFβ). The three centroids were evaluated in two publicly available datasets of prostate microdissected stroma (GSE26910, [9]) and CAFs (GSE6250, [14]). Other potentially interesting datasets were excluded due to unsuitable sample size or failure of quality control. For both suitable datasets, the processed data downloaded from the Gene Expression Omnibus data repository [47] were used. Similarity between our centroids and tumor-stroma/CAFs expression profile for the same genes was evaluated and results plotted in a heatmap.

### Experimental models

Human PCa cells (PC3) were purchased from the European Collection of Cell Cultures (ECACC) and maintained at 37°C/5% CO_2_ in DMEM medium supplemented with 10% fetal bovine serum. Human prostate fibroblasts, HPFs and CAFs, were isolated from healthy and intratumoral regions, respectively, of the prostate of PCa-bearing patients (Gleason score 4+5). Tissue samples were obtained aseptically from patients undergoing radical prostatectomy, upon informed consent in accord with the Ethics Committee of Fondazione IRCCS Istituto Nazionale dei Tumori di Milano. Tissues were digested overnight in DMEM supplemented with 5% fetal bovine serum and 1× Collagenase-Hyaluronidase Solution (STEMCELL™ Technologies Inc.). Cell suspension was centrifuged at 1,500 g for 5 minutes. The resulting fibroblast-rich pellet was suspended and plated in DMEM containing 10% fetal bovine serum and 4 mM L-Glutamine. Expression of E-cadherin and cytokeratin was assessed to exclude epithelial contamination of either HPFs or CAFs. HPFs and CAFs were maintained in culture for 6 passages after tissue isolation and then used for the analysis.

### Preparation of conditioned media

CM from HPFs, activated-HPFs, 133b-HPFs and CAFs were obtained from cells grown to sub-confluence, then serum starved for 48 h. CM were then harvested, clarified by centrifugation, and used freshly. Exosomes were isolated from 10 ml of culture media of either 48 h-serum starved HPFs or CAFs by ExoQuick-TC™ (System Biosciences, SBI), according to manufacturer's instruction.

### Fibroblast activation

HPFs were grown to sub-confluence and treated for 48 h with 10 ng/ml rTGFβ1 or 50 ng/ml IL6. Fresh serum-free medium was added for an additional 24 h before collection of CM.

### Transfection

miR-133b mimic and negative control (miR-Neg) were purchased as mirVana^®^ miRNA mimic molecules (Life Technologies, Carlsbad, CA, USA). Two siRNAs targeting *DROSHA* mRNA were designed: siDROSHA-1 (5′-AACGAGUAGGCUUCGUGACUU-3′) and siDROS HA-2 (5′-AAGGACCAAGUAUUCAGCAAG-3′). A non-targeting siRNA (siCTR, 5′-GCAUACAAUGGAGUUGU UA-3′) was used as control. Cells seeded at the appropriate density were transfected for 4 h at 37°C with 20 nM miRNA mimic or siRNA using Lipofectamine-2000 (Life Technologies), according to the manufacturer's instruction, and processed at different time intervals.

### RNA extraction

Total RNA from cells was extracted using TRIzol^®^ reagent (Life Technologies), according to the manufacturer's instructions. miRNA from CM was isolated using miRNeasy Kit (Qiagen, Hilden, Germany). Briefly, before miRNA extraction, CM was collected and sequential centrifugation was performed. Clarified-CM was first centrifuged at 14,000 g for 10 minutes, and then centrifuged at 1,000 g for 5 minutes. 350 μl of clarified and centrifuged CM was mixed with 1.6 pmol of spike-in non-human synthetic miRNA (mirVana miRNA mimic ath-miR-159a), providing an internal reference for normalization of technical variations between samples. Then, RNA was extracted using miRNeasy Kit, according to the manufacturer's instructions.

### miRNA and gene expression analysis

Quantification of miR-1, miR-133b, miR-143, miR-590-5p, miR-210, pri-miR-133b and pri-miR-210 expression levels was assessed by qRT-PCR using the following TaqMan^®^ microRNA expression assays (Life Technologies): miR-1, 002222; miR-133b, 002447; miR-143, 002249; miR-590–5p, 001984; miR-210, 000512; primary miR-133b, Hs03303651_pri; primary miR-210, Hs03302948_pri. mRNA expression was measured by qRT-PCR using the following Taqman^®^ gene expression assays: *ACTA2*, Hs00426835_g1; *DROSHA*, Hs00203008_m1; *FAP*, Hs00990806_m1; *S100A4* Hs00243202_m1; *COL4A2*, Hs00266237_m1. Amplifications were run on the 7900HT Fast Real-Time PCR System. Data were analyzed by SDS 2.2.2 software (Life Technologies) and reported as relative quantity with respect to a calibrator sample using the 2^−ΔΔCt^ method. *RNU48* (PN4427975) and *GAPDH* (PN4326317E) were used as normalizers for miRNAs and pri-miRNAs/mRNA expression, respectively. Spike-in miRNA ats-miR-159a (000338) was used as normalizer for miRNAs from CM.

### Immunofluorescence analysis

Cells were seeded on μ-Slide 8 well chamber (IBIDI GmbH, München, Germany), fixed in 4% formaldehyde/PBS for 15 min and incubated in methanol/acetone or 70% cold ethanol for 15 min at room temperature. Cell were probed with anti alpha-SMA (A2547; Sigma-Aldrich, St. Louis, MO, USA), anti-FAP (sc-65398; Santa Cruz Biotechnology, Santa Cruz, CA, USA), anti-S100A4 (ab27957; Abcam, Cambridge, UK), anti-collagen I (ab34710; Abcam), anti-3meH3K9 (ab8898; Abcam) or anti-gamma H2A.X (ab11174; Abcam) primary antibodies for 1 h and Alexa fluor488-labeled or Alexa Fluor594-labeled (Life Technologies) secondary antibodies for 1 h at room temperature. Nuclei were counterstained with 0.1 ug/ml of 4′, 6-diamindino-2-phenylindole (DAPI, Life Technologies). Images were acquired using a Nikon Eclipse E600 microscope by ACT-1 software (Nikon corporations, Tokyo, Japan) and processed by ImageJ.

### Immunoblotting analysis

Cells were lysed for 30 min on ice in lysis buffer (10 mM Tris–HCl, pH 7.4, 150 mM NaCl, 1% Triton X-100, 1 mM phenylmethanesulphonyl-fluoride, 5 μg/ml aprotinin, 20 μg/ml leupeptin). Lysates were clarified by centrifugation, and twenty μg of protein extracts were fractionated by SDS-PAGE and transferred onto nitrocellulose using standard protocols. Equal protein loading was verified by Ponceau staining. Filters were blocked in PBS 1X Tween-20 with 5% of skim milk and incubated overnight with primary specific antibodies for α-SMA (A2547; Sigma-Aldrich), FAP (sc-65398; Santa Cruz Biotechnology), p21 (ab7960; Abcam), p16 (ab7962; Abcam) and Vinculin (V9131; Sigma-Aldrich). The filters were then incubated with the secondary peroxidase linked whole antibodies. Bound antibody was detected using the Novex ECL, HRP Chemiluminescent substrate Reagent Kit (Life Thecnologies).

### Statistical analysis

Data are presented as mean values ± SD from at least three independent experiments. Statistical analysis of the data was performed by two-tailed Student's *t* test. *P*-values < 0.05 were considered statistically significant. Statistics applied to microarray analyses is described in the relative sections.

## SUPPLEMENTARY FIGURES AND TABLES


