# Supplementary material for: Integrated gene and miRNA expression analysis of prostate cancer associated fibroblasts supports a prominent role for interleukin-6 in fibroblast activation
Source: Oncotarget. 2015 Sep 8;6(31):31441–60. doi: 10.18632/oncotarget.5056 (PMC4741617; doi:10.18632/oncotarget.5056)
Supplement: Supplementary file 1 [file oncotarget-06-31441-s001.pdf]

SUPPLEMENTARY FIGURES AND TABLES

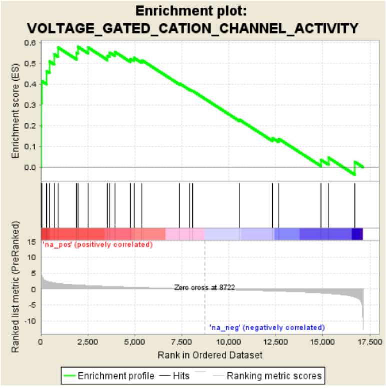

CACNA1H
KCNS3
SCN2A
CACNB1
CACNB3
SCN1B
KCNH2
CACNB2
KCNQ2

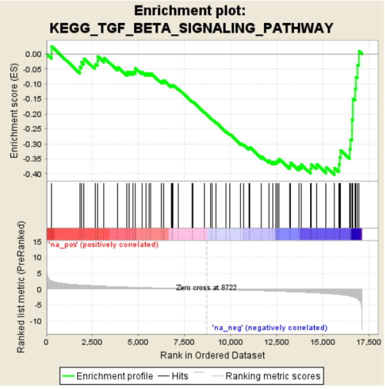

ACVRL1
TGFBR2
BMP6
THBS4
ID1
SMAD6
SMAD9
PPP2CA
SMURF2
ZFYVE16
ID2
BMP4
BMP2
SMAD5
BMPR2
BMPR1A
SMAD7
E2F4

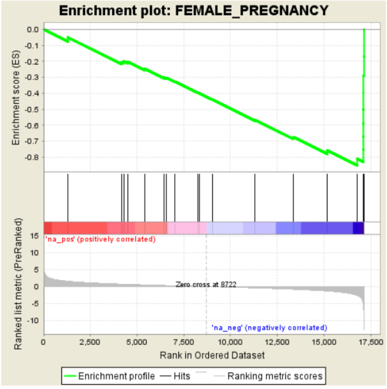

PSG5
PSG4
PSG3
PSG7
ADM
PSG9
PSG6
PSG1
OXTR

Supplementary Figure S1: Selected gene sets enriched in CAF-HPF comparison.

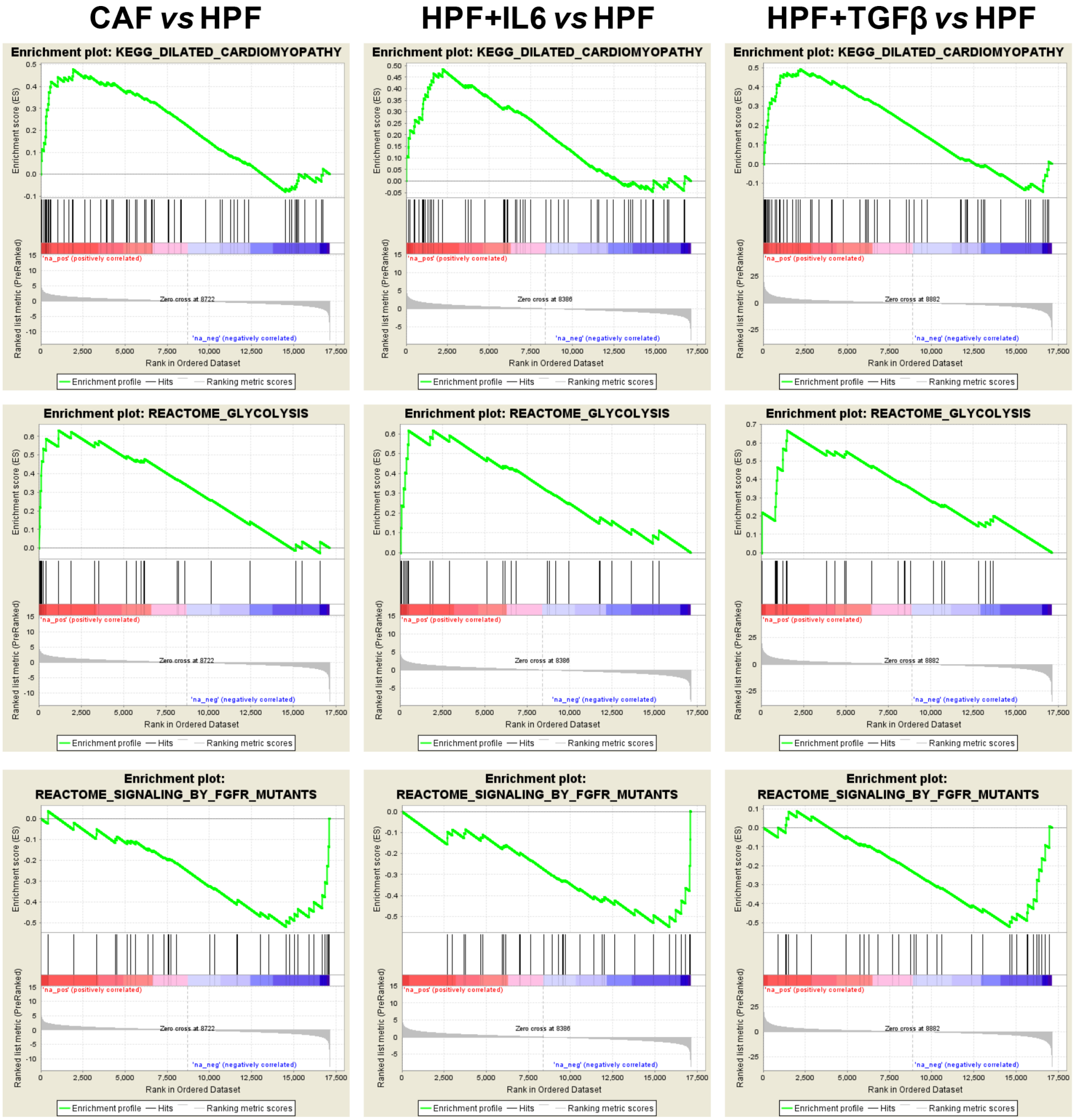

Supplementary Figure S2: Selected gene sets enriched in activated vs normal prostate fibroblasts. Examples of gene sets coherently enriched in all activated fibroblasts.

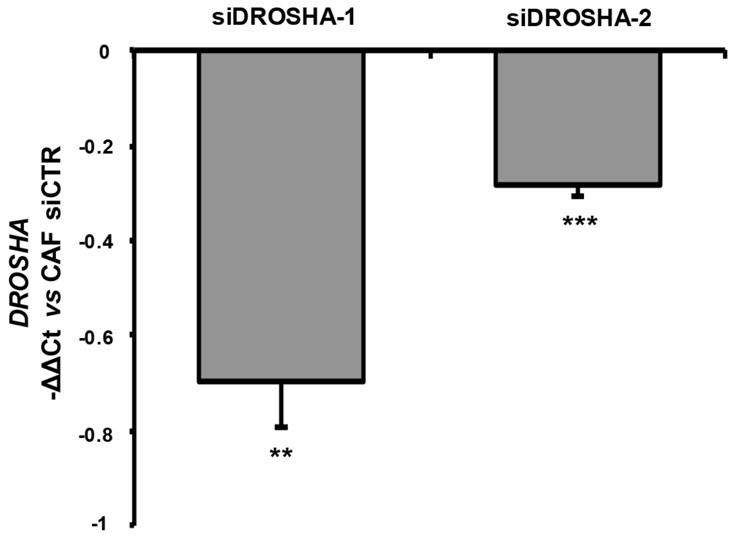

Supplementary Figure S3: Evaluation of the efficacy of siRNAs directed against *DROSHA* mRNA. *DROSHA* mRNA expression levels evaluated by qRT-PCR in CAFs transfected with two specific siRNAs (siDROSHA-1 and siDROSHA-2). Data are reported as −ΔΔCt with respect to cells transfected with control siRNA (siCTR).

Supplementary Table S1: Intersection of leading edge genes from the enriched gene sets of the cytoskeleton-muscle contraction network in CAF vs HPF comparison

Gene symbol	Frequence	Description
MYL9	10	myosin, light chain 9, regulatory
VCL	10	vinculin
DMD	7	dystrophin
MYL6B	7	myosin, light chain 6B
RAC1	7	ras-related C3 botulinum toxin substrate 1 (rho family, small GTP binding protein Rac1)
CFL1	6	cofilin 1
EZR	6	ezrin
MYL2	6	myosin, light chain 2, regulatory
MYLK	6	myosin light chain kinase
SORBS1	6	sorbin and SH3 domain containing 1
TNNT2	6	troponin T type 2
TPM4	6	tropomyosin 4
ACTG1	5	actin, gamma 1
ARPC1A	5	actin related protein 2/3 complex, subunit 1A

Supplementary Table S2: Representative genes differentially expressed in activated fibroblasts vs HPFs

TargetID	CAF		IL6		TGFbeta	
	logFC	*P*-Value	logFC	*P*-Value	logFC	*P*-Value
ACTA2	0.812383	*0.012522*	0.627006	*0.024348*	0.776291	*0.000128*
TGFB1	0.129996	*0.311275*	0.035071	*0.810537*	0.458302	*0.008598*
TGFB2	−0.15656	*0.614074*	−0.01131	*0.965815*	0.633925	*0.000871*
TGFB3	0.73943	*0.042833*	0.730303	*0.026377*	−0.84186	*0.002376*
MMP1	−2.06698	*0.104923*	−2.09203	*0.085617*	0.495947	*0.007103*
MMP2	−0.41597	*0.116991*	−0.54435	*0.040561*	1.474905	*1.1E-06*
MMP10	−1.25253	*0.190412*	−1.08227	*0.238905*	1.223799	*6.15E-05*
MMP14	0.187857	*0.293713*	0.13707	*0.400615*	0.706806	*0.000145*
MMP23A	0.712639	*0.123037*	0.727446	*0.149864*	0.699523	*0.008836*
MMP11	0.492123	*0.018596*	0.507052	*0.024427*	0.057089	*0.641004*

FC: fold-change
